# Dual-channel ultrasonic images empowered deep learning: significantly improving prediction of occult central lymph node metastases in solitary papillary thyroid microcarcinoma

**DOI:** 10.2478/raon-2026-0006

**Published:** 2026-02-06

**Authors:** Meihua Li, Chao Jia, Gang Li, Qiusheng Shi, Long Liu

**Affiliations:** Department of Ultrasound, Sijing Hospital of Songjiang District, Shanghai, China; Department of Ultrasound, Shanghai General Hospital, Shanghai Jiao Tong University School of Medicine, Shanghai, China

**Keywords:** papillary thyroid microcarcinoma, central lymph node metastasis, deep learning, ultrasound

## Abstract

**Background:**

Central lymph node metastasis (CLNM) significantly elevates the risk of postoperative recurrence and contributes to ongoing debates regarding the necessity of prophylactic dissection in clinically node-negative papillary thyroid microcarcinoma (PTMC). Therefore, accurate preoperative prediction of occult CLNM is crucial for tailoring individualized treatment strategies.

**Patients and methods:**

This retrospective study included 461 patients with PTMC from two hospitals who underwent preoperative ultrasound. A dual-channel deep learning (DL) model was developed by combining longitudinal and transverse ultrasound images. The model’s performance was compared with single-direction DL models and a clinical model using machine learning classifiers. Performance was evaluated using the area under the receiver operating characteristic curve (AUC) and calibration curves.

**Results:**

The dual-channel DL model outperformed the single-direction models, with AUC values of 0.765 in the training set and 0.726 in the external test set. The combined model, which integrated DL features and clinical indicators, achieved the highest AUC of 0.900 in the training set and 0.873 in the external test set, surpassing both the deep learning model using fused DL model (DL_F) and clinical models.

**Conclusions:**

The dual-channel DL model demonstrated superior performance in predicting occult CLNM in PTMC patients. When combined with clinical features, it offers a robust tool for personalized risk stratification and treatment decision-making, providing a non-invasive method for predicting occult CLNM and supporting individualized treatment strategies.

## Introduction

Papillary thyroid carcinoma is the most prevalent malignant tumor in the endocrine system.^1^ Over the past decade, its incidence has been rising significantly.^2^ Papillary thyroid microcarcinoma (PTMC) is a special subtype of papillary thyroid carcinoma. It has a maximum diameter of no more than 1 centimeter and makes up 60%–90% of newly diagnosed papillary thyroid carcinoma (PTC) cases. While the overall 10-year survival rate for PTMC patients is relatively high, about 13.5%–35.6% of them experience cervical lymph node metastasis.^3–5^ Among those with cervical lymph node metastasis, roughly 85% present with central lymph node metastasis (CLNM). This pathological manifestation significantly elevates the risk of local recurrence after surgery^6^, thereby influencing clinical treatment decisions and prognostic management.

The role of prophylactic central lymph node dissection in clinically node-negative (cN0) PTMC remains contentious globally, with persistent divergences in risk-benefit evaluations among surgical societies.^7–9^ Recent clinical developments have introduced non-surgical alternatives (active surveillance and thermal ablation) for managing low-risk thyroid cancers. While these approaches show favorable prognoses with preserved organ function, 2.1% to 2.6% of patients ultimately develop CLNM, highlighting the need for refined risk assessment.^10^ This clinical progression frequently necessitates additional surgical interventions and potentially adjuvant radioactive iodine therapy, which may significantly compromise both physical and psychological well-being in affected individuals.

Precision risk stratification for occult CLNM represents a critical determinant in personalizing PTMC management. Current prediction algorithms primarily combine ultrasound imaging features with clinical indicators; however, they still face significant challenges in predictive accuracy due to interobserver variability in image interpretation and biological heterogeneity.^11^ The exponential growth of artificial intelligence in medical imaging has catalyzed the application of DL architectures for CLNM prediction in PTMC.^12^ However, the current body of evidence highlights a critical gap in the availability of rigorously validated artificial intelligence based predictive frameworks specifically tailored to detect occult CLNM.

In this study, we have developed an innovative deep neural network model. This model is constructed using dual-channel fused ultrasound images, incorporating both transverse and longitudinal sections, and is further integrated with clinical characteristic data. The primary objective of this model is to provide accurate preoperative predictions of occult CLNM in patients with PTMC. By achieving this, the model aims to support decision-making processes in selecting individualized treatment strategies for PTMC patients.

## Materials and methods

### Study samples

This retrospective multicenter study was conducted in accordance with the ethical guidelines of the Declaration of Helsinki and received approval from the Ethics Committee of Shanghai General Hospital (Number, 2023417). Due to its retrospective design, informed consent from patients was not required. Patients with PTMC were consecutively recruited from two hospitals between January 2017 and December 2024. All participants underwent preoperative ultrasound examinations, and their diagnoses were confirmed by postoperative pathology. Exclusion criteria included patients without complete ultrasound imaging data and those with pre-surgical confirmation of cervical lymph node metastasis via fine-needle aspiration.

### Indications and extent of surgery for papillary thyroid microcarcinoma

For patients with PTMC, surgical intervention was indicated for those with a Bethesda V or VI classification confirmed by fine-needle aspiration, as well as for patients who declined active surveillance after providing informed consent. The extent of surgery was determined based on tumor characteristics and risk stratification. Patients without high-risk features were managed with a thyroid lobectomy, which involved resection of the affected lobe, pyramidal lobe, and isthmus, along with ipsilateral central lymph node dissection. A total thyroidectomy, encompassing the resection of both thyroid lobes, the pyramidal lobe, and the isthmus, in addition to bilateral central lymph node dissection, was conducted for patients presenting with bilateral multifocal tumors, isthmus tumors exhibiting extrathyroidal extension, gross extrathyroidal extension, vascular invasion, a history of head or neck radiation exposure during adolescence, a familial history of non-medullary thyroid cancer, high-risk PTMC subtypes, high-risk stratification, or a necessity for postoperative radioactive iodine therapy. Central lymph node dissection encompassed the excision of prelaryngeal, pretracheal, and ipsilateral paratracheal lymph nodes.

### Clinical indicators

This study included 13 clinical indicators: age, sex, tumor location, size, aspect ratio, extrathyroidal extension, echogenicity, margin, shape, vascularity, calcification, capsular contact, and discontinuous capsule. The definitions and measurement methods for each of these clinical indicators are detailed in the appendix (Table 1).

**TABLE 1.** Characteristics of the distribution of clinical data of patients among groups

Clinical indicators	Training set (n = 265)	Validation set (n = 114)	External test set (n = 82)	Statistics	*P* value
Age (years) ^a^	43.0 (35.0–52.0)	45.5 (35.3–54.0)	45.0 (34.0–53.0)	0.401	0.818
Size (mm) ^a^	6.1 (4.7–7.8)	5.6 (4.2–7.0)	6.0 (4.6–7.3)	4.090	0.129
Aspect ratio ^a^	1.1 (0.9–1.3)	1.0 (0.9–1.2)	1.1 (0.9–1.3)	3.547	0.170
Sex				1.493	0.474
Male	69 (26.0)	24 (21.1) ^b^	23 (28.1) ^b^		
Female	196 (74.0)	90 (79.0)	59 (72.0)		
Location				15.632	0.016
Upper segment	33 (12.5)	15 (13.2)	22 (26.8) ^b^		
Middle segment	146 (55.1)	58 (50.9)	38 (46.3)		
Lower segment	57 (21.5)	29 (25.4)	20 (24.4)		
Isthmus	29 (10.9)	12 (10.5)	2 (2.4)		
ETE				4.733	0.094
Absence	226 (85.3)	102 (89.5)	77 (93.9)		
Presence	39 (14.7)	12 (10.5)	5 (6.1)		
Echogenicity				4.110	0.391
Hyperechoic/isoechoic	11 (4.2)	8 (7.0)	5 (6.1)^b^		
Hypoechoic	200 (75.5)	75 (65.8)	59 (72.0)		
Markedly					
Hypoechoic	54 (20.3)	31 (27.2)	18 (22.0)		
Margin				2.449	0.294
Clear	113 (42.6)	53 (46.5)	29 (35.4)		
Unclear	152 (57.4)	61 (53.5)	53 (64.6)		
Shape				5.242	0.073
Regular	115 (43.4)	47 (41.2)	24 (29.3)		
Irregular	150 (56.6)	67 (58.8)	58 (70.7)		
Vascularity				5.642	0.465
Type I	95 (35.9)	38 (33.3)	24 (29.3)		
Type II	21 (7.9)	9 (7.9)	12 (14.6)		
Type III	140 (52.8)	63 (55.3)	41 (50.0)		
Type IV	9 (3.4)	4 (3.5)	5 (6.1)		
Calcification Absence	123 (46.4) ^b^	58 (50.9)	41 (50.0)	2.902	0.821
Microcalcification	100 (37.7)	38 (33.3)	25 (30.5)		
Coarse type	25 (9.4)	13 (11.4)	10 (12.2)		
Mixed type	17 (6.4)	5 (4.4)	6 (7.3)		
CC				0.212	0.900
Absence	97 (36.6)	41 (36.0)	32 (39.0)		
Presence	168 (63.4)	73 (64.0)	50 (61.0)		
DC				0.496	0.780
Absence	186 (70.2)	83 (72.8)	56 (68.3)		
Presence	79 (29.8)	31 (27.2)	26 (31.7)		

1 Except for the special markings, the numbers in the table represent the number of patients, and the numbers in parentheses represent the percentage of the number of patients within the group.

a indicates that the values in the table are expressed as the median and interquartile range. The Kruskal-Wallis test is used for the comparison among groups.

b indicates that the subgroup percentages do not sum to 100% due to rounding to one decimal place.

1 CC = capsular contact; DC = discontinuous capsule; ETE = extrathyroidal extension

### Method for obtaining ultrasound images

Thyroid ultrasound images were acquired using the Aplio 500 or Aplio i900 (Canon Medical Systems, Tokyo, Japan) with high-frequency linear array probes, operating at 7–14 MHz for the Aplio 500 and 5–18 MHz for the Aplio i900. Detailed information on the ultrasound examination and thyroid nodule measurement protocols can be found in the appendix.

### Image processing and establishment of the deep learning model

**FIGURE 1.**
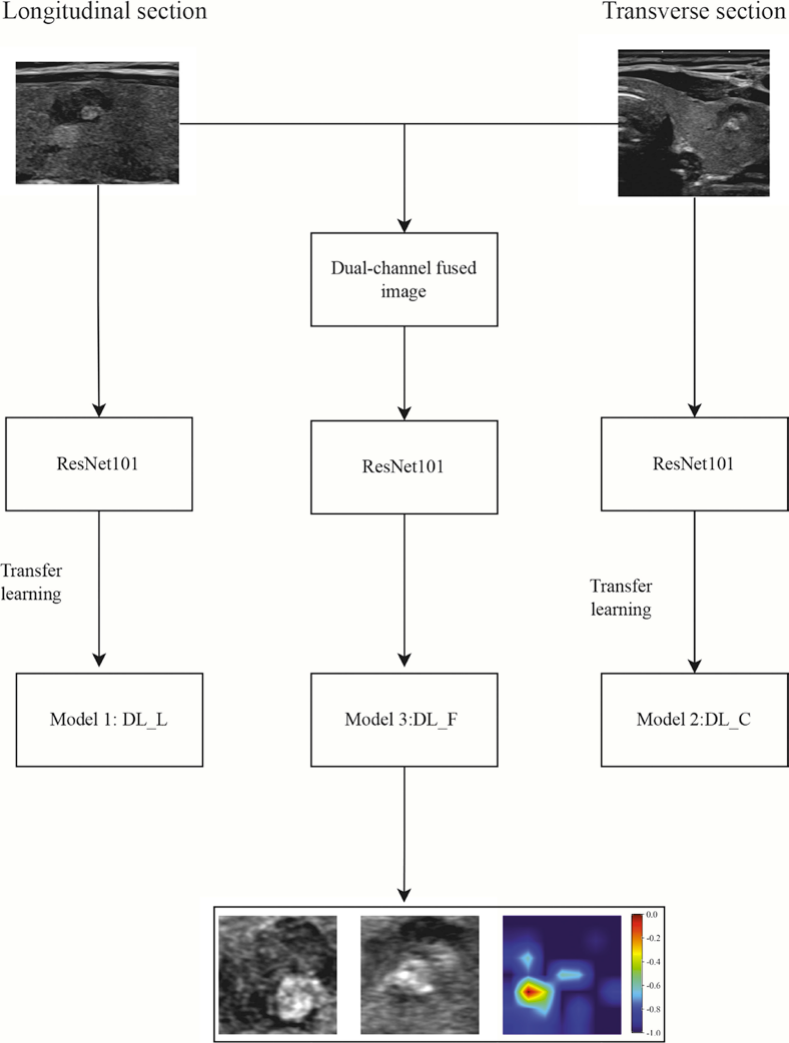
Flowchart depicting the construction of the dual-channel deep learning model. The model comprises two components, Model 1 and Model 2, which are built using ultrasonic images from the longitudinal and transverse sections of the thyroid tumor, respectively. These images are then fused to create dual-channel images. The ResNet101 architecture is employed to train the fused model, referred to as DL_F (Model 3). DL_C = cross-sectional deep learning model; DL_F = deep learning model using dual-channel fused images; DL_L = longitudinal deep learning model

First, the ultrasound images of the thyroid tumor’s largest section were selected for training the DL neural network. Since these maximum-section images consist of 2D images in two directions – the longitudinal and transverse sections – we trained two separate convolutional neural networks: Model 1, the longitudinal DL model (DL_Ll), and Model 2, the cross-sectional DL model (DL_C). Both DL models were based on ResNet101, and we loaded pre-trained ImageNet weights into the 2D DLmodels. In addition, we developed a new model, Model 3: fused DL model (DL_F), which simultaneously utilizes information from both the longitudinal and transverse sections. This model, trained with dual-channel fused images containing tumor information from both sections, is referred to as a dual-channel DL model. The structure of this dual-channel model is presented in Figure 1, and the detailed construction process is described in the appendix. The relevant code is available at the following website (https://github.com/lzlcsk2024/Dual-channel-.git). To better understand the model’s feature recognition and decision-making mechanisms during data processing, we employed the gradient-weighted class activation mapping method to perform a visual analysis of Model 3.^13^

### Development of the combined model

In this study, we first extracted DL features from the fused images. These features were then combined with clinical features and standardized. Subsequently, relevant features were selected based on their *P* values, and the *Least Absolute Shrinkage and Selection Operator* method was utilized for dimensionality reduction. The remaining features were used to construct the Model 4 (combined model) using machine learning. Three classifiers were employed: Logistic Regression (LR), Light Gradient Boosting Machine (LightGBM), and Naive Bayes. The classifier with the highest area under the receiver operating characteristic curve (AUC) value was selected as the output of the fused model, which became the Combined model. To further analyze the machine learning algorithm of the combined model, we conducted a visual analysis using SHAP (Shapley Additive ex-Planations). The detailed construction process of the combined model is provided in the appendix.

### Clinical model

A clinical model (Model 5) was developed using all clinical indicators, with classifiers such as LR, LightGBM, and Naive Bayes. The construction process of the clinical model followed the same approach as that of the combined model.

### Definition of occult central lymph node metastasis

Occult CLNM is defined as the absence of CLNM detected on preoperative ultrasound or clinical evaluation, with subsequent pathological confirmation of central CLNM following surgery.^14^

### Statistical analysis

Statistical analysis was performed using SPSS 21.0 software (SPSS Inc., Chicago, Illinois, USA) and python programming language (version 3.7). Continuous variables are presented as median and interquartile range, while categorical data are shown as the number of cases and percentages. For group comparisons, the Kruskal-Wallis test or chisquare test was used, depending on the data type. The AUC was calculated to assess model performance. All models were evaluated using the validation set and then tested on an independent external test set. The Delong method was applied to compare AUC values between models. The calibration curve was used to assess the agreement between predicted and actual probabilities. A *P* value of less than 0.05 was considered statistically significant.

## Results

**FIGURE 2.**
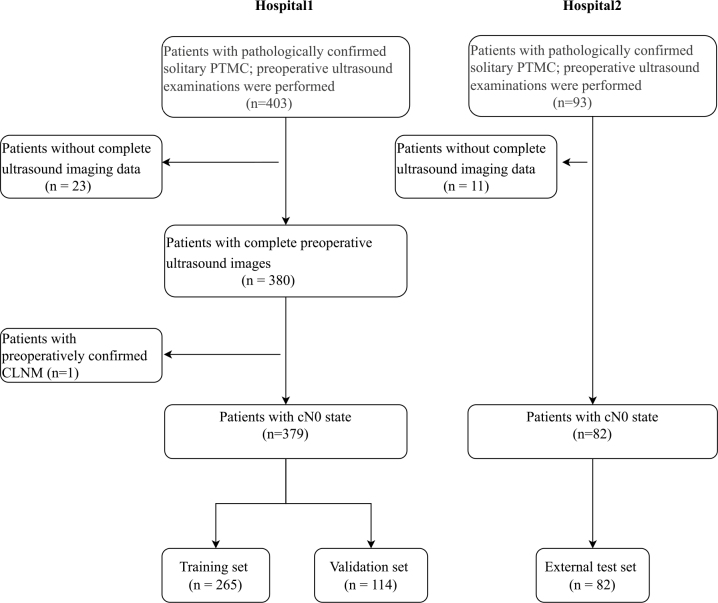
Flowchart illustrating the patient enrollment process. PTC = papillary thyroid carcinoma; PTMC = papillary thyroid microcarcinoma; CLNM = cervical lymph node metastasis

A total of 496 patients were initially selected for this study. However, 34 patients were excluded due to incomplete ultrasound image data, and 1 patient was excluded due to confirmed CLNM before surgery. According to the research design and criteria, these 35 patients were excluded (the specific exclusion process is shown in Figure 2). Finally, 461 patients were included in the study. The average age of the patients was 44.5 ± 11.6 years, with females comprising 74.8% (345/461) and males comprising 25.2% (116/461). In total, 922 ultrasound images were used in the study. Among these, 758 images from 379 patients were allocated to the training and validation sets, which were used for model development and internal validation. Additionally, 164 images from 82 patients were used for external testing to assess the model’s generalization ability. Statistical analysis revealed no significant differences in clinical indicators between the training set, validation set, and external test set (see Table 1 for details), with *P* values ranging from 0.016 to 0.900.

### Performance of the Dual-channel Model

**FIGURE 3.**
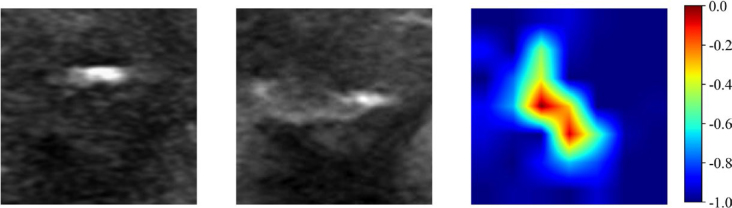
Gradient-weighted Class Activation Mapping images predicted by the dual-channel deep learning model. The left image represents the minimum bounding rectangle containing the largest section of the tumor on the longitudinal section. The middle image represents the minimum bounding rectangle containing the largest section of the tumor on the transverse section. The right image represents the Gradient-weighted Class Activation Mapping image.

In both the training and validation sets, the AUC values of Model 3: DL_F were 0.765 (95% confidence interval [CI]: 0.703 - 0.826) and 0.723 (95% CI: 0.617 - 0.828), respectively. These values were significantly higher than those of Model 1: DL_L (0.673 [95% CI: 0.603 - 0.742] and 0.671 [95% CI: 0.563 - 0.779], *P* = 0.049, 0.019) and Model 2: DL_C (0.618 [95% CI: 0.542 - 0.693] and 0.542 [95% CI: 0.426 - 0.658], *P* = 0.002, 0.042). In the external test set, the AUC value of Model 3 was 0.726 (95% CI: 0.574–0.878), which was also higher than the AUC values of Model 1 (0.547 [95% CI: 0.400-0.691]) and Model 2 (0.510 [95% CI: 0.359-0.662], *P* = 0.043, 0.049). Additionally, gradient-weighted class activation mapping (Grad-CAM) was used to perform an interpretability analysis of Model 3 (Figure 3).

### Performance of the clinical model

The LR classifier demonstrated relatively high AUC values in both the training set and validation set, with values of 0.720 (95% CI: 0.656–0.784) and 0.704 (95% CI: 0.588–0.819), respectively. As a result, it was selected as the final classifier for the clinical model (Model 5: Clinical Model). The AUC value for Model 5 in the external test set was 0.671 (95% CI: 0.523–0.818).

### Establishment and performance evaluation results of the combined model and model visualization

**FIGURE 4.**
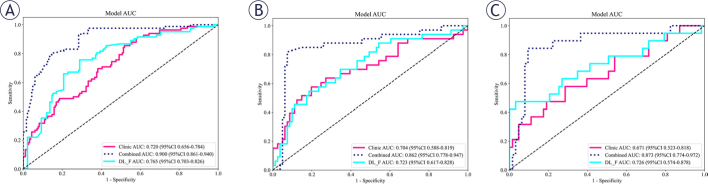
The AUC values of the combined model. Compared with the clinical model and the dual-channel model, the combined model has higher AUC values on the training set **(A)**, the validation set **(B)**, and the external test set **(C)**. AUC = area under the receiver operating characteristic curve; CI = confidence interval; LightGBM = Light Gradient Boosting Machin; LR = logistic regression

For the fused dual-channel images, this study extracted 2048 DL features, from which 64 compressed features were obtained and integrated with all clinical indicators. After multiple rounds of feature screening, 7 DL features and 4 clinical features (age, gender, nodule size, aspect ratio) were retained. The LightGBM classifier demonstrated higher AUC values in both the training set (0.900 [95% CI: 0.861–0.940]) and the validation set (0.862 [95% CI: 0.778–0.947]) compared to other classifiers and was selected as the final model for the combined model (Model 4). The AUC value of Model 4 in the external test set was 0.873 (95% CI: 0.774–0.972) (Figure 4).

**FIGURE 5.**
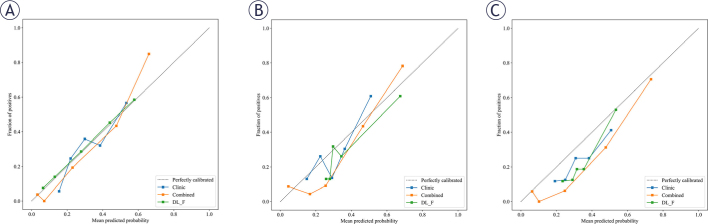
Calibration curves of different models. **(A)** Calibration curves of the combined model, dual-channel model and the clinical model in training dataset; **(B)** Calibration curves in the validation dataset; **(C)** Calibration curves in the external test set.

**FIGURE 6.**
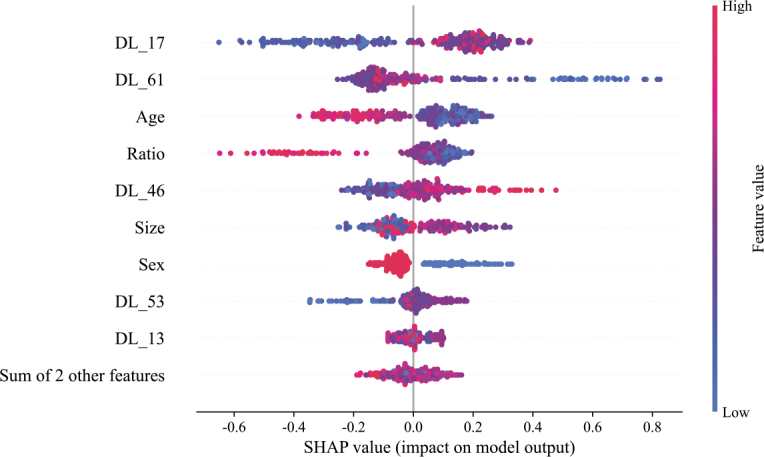
Contributions of individual features in the combined model. The summary plot displays the distribution of SHAP values for each feature across all predictions in the training cohort, providing a comprehensive overview of feature importance and their respective impacts. SHAP = SHapley Additive exPlanations

In both the training and validation sets, the AUC of Model 4 was significantly higher than that of Model 3 and Model 5 (training set: all *P* < 0.001; validation set: Model 4 vs. Model 3, *P* = 0.042; Model 4 vs. Model 5, *P* = 0.012). In the external test set, the AUC of Model 4 was higher than that of Model 5 (*P* = 0.013). However, the difference between Model 4 and Model 3 was not statistically significant (*P* = 0.122). Calibration curves indicated that the prediction performances of Model 3, Model 4, and Model 5 were all satisfactory (all *P* values > 0.05) (Figure 5). The SHAP value graph illustrated the contributions of DL features and clinical features to Model 4 (Figure 6).

## Discussion

The objective of this study was to develop an AI model for preoperative prediction of occult CLNM in PTMC. A retrospective analysis of 922 ultrasound images from 461 patients with PTMC was conducted, employing DL and various machine learning techniques to build the AI prediction model. Key findings from this research demonstrated that the dual-channel model outperformed the individual longitudinal-axis and transverseaxis DL models in predictive performance. Furthermore, its accuracy was further enhanced when integrated with clinical features. These findings hold significant clinical implications, as the model can aid physicians in developing more personalized treatment and management strategies for patients with low-risk thyroid cancer, thereby improving patient prognosis and reducing unnecessary medical resource utilization.

In this study, we observed that the AUC value of Model 3 was significantly higher than those of Models 1 and 2 across all datasets. Specifically, when compared to individual DL models based on transverse and longitudinal sections, the dual-channel model demonstrated superior performance in predicting occult CLNM. The dualchannel model integrates both transverse and longitudinal ultrasound image data of the tumor. Its unique dual-channel architecture allows for efficient extraction and fusion of features across multiple dimensions, enabling comprehensive capture of imaging characteristics associated with occult CLNM. This capability is central to its enhanced predictive performance. In contrast to previous studies, which predominantly focused on ultrasound images from a single section, the dual-channel model used in this study introduces a novel perspective and approach to the field.^12,15^ These findings not only expand the technical methods for predicting occult CLNM in patients with PTMC but also offer new insights and directions for future research. Clinically, the high AUC value of this model suggests that it can more accurately predict CLNM prior to surgery, facilitating more precise preoperative assessments and enabling the formulation of more tailored surgical strategies for patients.

In constructing the combined model for this study, we applied multiple feature dimensionality reduction methods, ultimately retaining four clinical features: age, gender, nodule size, and aspect ratio. SHAP value analysis revealed that younger patients, male patients, and those with larger nodules were more likely to experience occult CLNMs, which aligns with findings from numerous previous studies.^16,17^ In PTMC, younger patients and males are at a higher risk for occult CLNM, with tumor size widely recognized as a key factor influencing this process. The underlying mechanism is attributed to the enhanced proliferative and invasive phenotypes of tumor cells in younger patients, thereby enabling more efficient invasion of local tissues and penetration of the lymphatic vasculature, ultimately culminating in lymph node metastasis. Additionally, male hormone levels and immune status may play a role in tumor development and metastasis, making men more prone to metastasis. Larger nodules are also more likely to invade surrounding lymphatic and blood vessels, providing greater opportunities for metastasis. This study not only corroborates these existing conclusions but also, through rigorous feature dimensionality reduction and SHAP value analysis, provides a more robust quantitative foundation for understanding the significance of these factors in predicting metastasis. However, analysis of the SHAP value graph revealed that the relationship between the aspect ratio and SHAP value could not be determined due to considerable overlap between nodules with higher and lower aspect ratios among the patients with occult central lymph node metastasis.

In this study, we extracted DL features from dual-channel fused images and integrated them with clinical features to develop a combined model. This model demonstrated exceptional performance across different datasets, with its prediction results in both the training and validation sets significantly outperforming those of the single dualchannel model and the clinical model. In previous studies, Wang *et al*. employed a single DL model to predict occult CLNM in PTMC. Although this model successfully extracted image features, it lacked the incorporation of clinical factors, which hindered its ability to comprehensively and accurately predict metastasis risk, especially when considering diverse individual patient characteristics.^12^ In contrast, primarily relied on clinical features to construct a prediction model. While this approach accounted for overall patient condition, it was limited by its inability to extract latent information from imaging data.^18^ Similar to the clinical model in our study, this approach overlooked crucial imaging-level information when faced with complex and variable imaging manifestations, leading to suboptimal prediction performance. By contrast, our combined model cleverly combines the strengths of both approaches. It inherits the robust imaging feature extraction capability of the dual-channel model, which enables the detection of subtle features in ultrasound images. Additionally, by incorporating clinical features, it considers individual patient differences and disease progression characteristics, enabling a comprehensive, multi-level analysis of the patient’s condition. The integration of this multidimensional information allows the combined model to more accurately and comprehensively assess the risk of occult CLNM in PTMC patients across both the training and test sets. This significantly enhances the model’s prediction accuracy and reliability, providing clinicians with more precise and effective decision-making support during the diagnosis and treatment of PTMC. It is expected to play a key role in future clinical practice and contribute to the continued advancement of thyroid cancer diagnosis and treatment.

This study has several limitations. First, data were obtained from only two hospital centers, which may limit the generalizability of the findings. Second, the retrospective design introduces potential information bias due to incomplete or inaccurate medical records, complicating the control of confounding factors. Additionally, the AI model is not an end-to-end intelligent system; significant human intervention during the modeling process may introduce subjectivity. The dual-channel model integrates information from only two sections, limiting its ability to capture the full variability of thyroid tumors across multiple sections and volumetric dimensions, which may reduce predictive accuracy. Future research should focus on models incorporating multi-sectional or tumor volume data to better represent tumors and improve predictive efficiency.

## Conclusions

The DL model that integrates dual-channel ultrasound imaging information effectively predicts occult CLNM in patients with solitary PTMC.
